# Effectiveness of task-shifting for the detection of diabetic retinopathy in low- and middle-income countries: a rapid review protocol

**DOI:** 10.1186/s13643-020-01553-w

**Published:** 2021-01-04

**Authors:** Covadonga Bascaran, Nyawira Mwangi, Fabrizio D’Esposito, Iris Gordon, Juan Alberto Lopez Ulloa, Shaffi Mdala, Jacqueline Ramke, Jennifer R. Evans, Matthew Burton

**Affiliations:** 1 London School of Hygiene & Tropical Medicine, Keppel Street, London, WC1E 7HT UK; 2 Kenya Medical Training College, Nairobi, Kenya; 3 The Fred Hollows Foundation, Melbourne, Australia; 4 Centro Mexicano de Salud Visual Preventiva, Mexico, DF Mexico; 5 Queen Elizabeth Central Hospital, Blantyre, Malawi; 6 School of Optometry and Vision Science, University of Auckland, Auckland, New Zealand; 7 Moorfields Eye Hospital, London, UK

**Keywords:** Low- and middle-income countries, Diabetic retinopathy, Task-shifting, Effectiveness

## Abstract

**Background:**

Diabetic retinopathy is the most common ocular complication of diabetes and a cause of vision loss in adults. Diabetic retinopathy screening leading to early identification of the disease followed by timely treatment, can prevent vision loss in people living with diabetes. A key barrier to the implementation of screening services in low- and middle-income countries is the low number of ophthalmologists per million population. Interventions that shift screening to non-ophthalmology cadres have been implemented in programmes in low- and middle-income countries and are routinely used in high-income countries. The aim of this rapid review is to summarise the published literature reporting the effectiveness of task-shifting interventions for the detection of diabetic retinopathy by non-ophthalmologists in low- and middle-income countries.

**Methods:**

We will search MEDLINE, Embase, Global Health and Cochrane Register of Studies for studies reporting task-shifting interventions for diabetic retinopathy detection. The review will include studies published in the last 10 years in the English language. We will include any interventional or observational comparative study measuring outcomes in terms of participation or access to diabetic retinopathy detection services (uptake) and quality of diabetic retinopathy detection services (detection, severity, diagnostic accuracy). For included studies, cost-effectiveness of the task-shifting intervention will also be presented. Two reviewers will screen search results independently. The risk of bias assessment and data extraction will be carried out by one reviewer with verification of 10% of the papers by a second reviewer. The results will be synthesised narratively.

**Discussion:**

Differences in health systems organization, structure and resources will determine the need and success of task-shifting interventions for DR screening. The review will examine how these interventions have been used and/or tested in LMICs. The results will be of interest to policy makers and programme managers tasked with designing and implementing services to prevent and manage diabetes and its complications in similar settings.

**Systematic review registration:**

OSF: https://osf.io/dfhg6/.

## Background

Diabetic retinopathy (DR) is the most frequent microvascular complication of diabetes and a significant cause of vision loss in adults [1]. Vision loss due to DR can largely be prevented through regular retinal screening for timely identification of DR, followed by referral and treatment [2–5].

DR screening has been shown to be cost-effective when compared to no screening or opportunistic screening [3, 6, 7]. However, cost-effectiveness requires high coverage and this is challenging in low- and middle-income (LMICs) countries [8] where the available eye health services are clustered around the cities and access to them from the remote and rural populations is often difficult [9]. Few countries worldwide have been successful in establishing population-based screening programmes that reach most of the patients with diabetes [3]. The uptake of retinal screening depends on context-specific factors related to the service provider, the wider organization and structure of the health system and the patient [10–12].

A crucial limitation to deliver DR screening in LMICs is the low availability of ophthalmologists, particularly in sub-Saharan Africa (SSA), where the mean ophthalmologist density per million population is 3.7 [13]. In this context, it is not realistic to rely on ophthalmologists to examine the retinas of the growing population of people living with diabetes (PLWD) unless it is at the expense of the delivery of other critical eye care services. This is not only a challenge for LMICs, and programmes in high-income countries (HICs) have overcome this issue by successfully adopting task-shifting in DR screening by training retinal graders, thus freeing ophthalmologists time to perform other tasks [14]. The WHO defines task-shifting as the transfer of tasks to existing cadres of healthcare workers with shorter training and fewer qualifications or to newly created cadres who receive a competency-based training for the specific task [15]. There is some evidence of the successful use of task-shifting in SSA in other health fields including diabetes [16, 17]. However, it is not clear whether task-shifting interventions for the detection of DR in LMICs results in an increase in the uptake of screening and higher DR detection rate.

There are several factors that can potentially affect the effectiveness of task-shifting interventions for DR screening. Firstly, the choice, level and training of the cadre performing the screening is a key consideration and is likely to be different depending on the composition and competencies of the health workforce in a particular country. The quality of the screening is measured by the diagnostic test accuracy (DTA) of the method of choice in the hands of non-ophthalmologists [18]. It is recommended that population-based screening programmes should reach over 80% sensitivity and 95% specificity [6, 19]. Screening performed by photographers has been shown to be more accurate when done by photographers with specialist medical or eye care qualifications [20]. There is also evidence that non-medical retinal image graders can achieve the threshold level of sensitivity and specificity in both mydriatic and non-mydriatic retinal assessment [18].

Secondly, the screening technique used can also affect the DTA. The recommended technique is photography-based screening which adds the possibility of quality assurance [14]. DR screening programs in LMICs are gradually adopting retinal photography. However, in many clinics without cameras, other methods of retinal examination that are not necessarily effective as screening techniques are still used [18]. The sensitivity of direct ophthalmoscopy in detecting DR is low (65%) and indirect ophthalmoscopy requires extensive training and experience [21]. Slit lamp bio-microscopy has a high sensitivity and specificity but also requires significant training and the availability of a slit lamp. Optical coherence tomography (OCT) for the detection of diabetic maculopathy is expensive and rarely available outside tertiary level eye departments in LMICs.

Thirdly, the use of mydriasis can improve the effectiveness of task-shifting by increasing the number of gradable images [18]. However, non-mydriatic imaging presents several advantages for screening programmes, like reduced screening time, less inconvenience for patients and importantly no limitations for using cadres that may not be allowed to instil eye drops.

Finally, an important consideration in LMICS is the need to reach rural and remote populations with difficult access to eye health services. Outreach services delivered by non-ophthalmologists can be an answer to this problem, but must ensure that DTA is maintained [18, 20]. Evidence suggests that DTA is higher in hospital-based screening services compared with outreach services [15]. Several approaches for DR screening in outreach services that can lend themselves to task-shifting have shown promising accuracy levels. These include telemedicine using digital imaging, the use of artificial intelligence-supported cameras or smart phone-based technology for image capture [22–26].

This rapid review aims to identify and synthesise the peer reviewed literature on the effectiveness of task-shifting for the detection of DR in LMICs. It is an important topic given the predicted epidemic of diabetes mellitus which will disproportionally affect LMICs which will require major prevention strategies to prevent the corresponding increase in blindness caused by diabetic retinopathy. There is a body of evidence to support the effectiveness of task-shifting in DR screening in high-income settings which suggests that this may be a viable strategy in LMICs. However, given the differences in structure, organization and resourcing in health systems in LMICs, evidence from HICs may not be generalizable to these settings. The aim of this rapid review is to summarise the published literature reporting the effectiveness of task-shifting interventions for the detection of diabetic retinopathy by non-ophthalmologists low- and middle-income countries.

## Methods

### Rapid review question

Can task-shifting improve the detection of diabetic retinopathy (DR) compared with eye examination done by ophthalmologists among adults with diabetes in LMICs?

### Protocol and registration

A rapid review was chosen as it is an effective methodology to support health programme decision-making through quality evidence in a timely and cost-effective manner. These rapid review methods were informed by the WHO practical guide on rapid reviews and we used the STARR (Selecting Approaches for Rapid Reviews) Decision Tool to carefully take into account the possible limitations introduced by the choice of methodology [27, 28]. We provide the adapted Preferred Reporting Items for Systematic Reviews and Meta-Analysis Protocols (PRISMA-P) in Additional file 1. The registration details are provided in Additional file 2. We will document any future amendments to the protocol on the Open Science Framework (OSF) registration site [29] (Table 1).

**Table 1:** Eligibility criteria

Population	Adults (aged ≥ 18 years) with type 1 or type 2 diabetes in LMICs
Intervention	Task shifting for DR detection: assessment of the target population for the presence of DR performed by individuals other than ophthalmologists
Comparison	Assessment of the target population for the presence of DR performed by ophthalmologists
Outcomes	Uptake 1. Proportion of patients accessing detection services among the adult population with type 1 and type 2 diabetes Quality 2. Proportion of patients diagnosed with DR among those accessing detection services 3. Severity of DR at detection 4. Diagnostic accuracy of the task-shifting intervention for DR detection Cost-effectiveness 5. Cost-effectiveness of the task-shifting intervention to increase DR detection
Study design	Any interventional or observational comparative study

### Eligibility criteria

The review will focus on adults only as they represent the larger population group at risk of DR and are the target of population-based screening programmes. We will include any study that measures any of the uptake and quality outcomes described in the above table. For included studies, we will also collect data on cost-effectiveness of the task-shifting intervention where available.

We will only include peer-reviewed publications reporting studies conducted in LMICs, as classified by the World Bank [8] in the last 10 years, in the English language with available full reports.

The following exclusion criteria will be applied: firstly, reviews, editorials, case studies, conference abstracts; secondly, studies involving health education, training programmes or qualitative studies and thirdly, studies conducted in high income settings.

### Information sources

Following guideline of the STARR Decision tool, we consulted the review commissioners (FHF) and conducted scoping searches on PubMed to inform the decision on which information sources to include, balancing the scope and time available to do the review [30]. The Cochrane Eyes and Vision Information Specialist (IG) will develop the search strategy which will include MEDLINE, Embase, Global Health and the Cochrane Register of Studies. We will not search the grey literature as it was considered that the priority for this review is to summarise the peer-reviewed evidence of effectiveness of task-shifting interventions required to influence and change policy and practice.

### Search strategy

Search strategy is included in Additional file 3.

### Data management and selection process

Two reviewers will independently review each title and abstract using the online review management software (Covidence, Veritas Health Innovation, Melbourne, Australia. Available at www.covidence.org). Any conflicts will be discussed and resolved. Two reviewers will independently screen the full text articles of potentially relevant studies against the inclusion and exclusion criteria and any will discuss any differences to reach consensus. A summary of the study selection process will be presented in a PRISMA flow diagram.

### Risk of bias assessment

One reviewer will use SING critical appraisal checklists (https://www.sign.ac.uk) to assess the risk of bias for included studies. This will be presented in narrative form with the review findings.

### Data extraction

We will develop an Excel data extraction form which will be tested by two reviewers on three papers. One reviewer will conduct data extraction and a second reviewer will check 10% of the studies for accuracy. If more than 1% errors are found, another 10% will be checked (Table 2).

**Table 2:** Data items

Data categories	Example data items
Publication characteristics	
	Authors, title, publication date, journal citation
Study characteristics	
	Design
	Dates of data collection
Population characteristics	
	Country
	Population demographics (age, gender)
	Sample size
Intervention characteristics	
	Setting (rural/urban, community/hospital)
	Task shifting cadre
	Training (type and duration)
	Scope of the task (screening, treatment, both)
	Eye examination procedures (Mydriatic/ non mydriatic, ophthalmoscopy/photography)
	Technology (cameras, ophthalmoscope, slit lamp, OCT)
	DR classification (EDTRS, other)
	Referral criteria/guidelines (periodicity; immediate referral for severe cases; compliance tracking)
	Quality assurance (audit, retraining, CPD or any other measure for quality assurance)
Outcomes	
	1. Proportion of patients accessing detection services among the adult population with type 1 and type 2 diabetes
	2. Proportion of patients diagnosed to have DR among those accessing detection services
	3. Diagnostic accuracy of the task-shifting intervention for DR detection
	4. Severity of DR at detection
	5. Cost-effectiveness of the task-shifting intervention

### Data synthesis

As we anticipate to find heterogeneity in study design, interventions, methods and outcome reporting, we will summarise data narratively following the SWiM reporting guidance: Synthesis Without Meta-analysis reporting items [31]. We will use tables to present the scope and nature of the evidence and descriptive statistics and visual displays to present effect estimates. We will use the GRADE (Grading of Recommendations, Assessment, Development and Evaluations) approach to assess the certainty of the evidence for the outcomes of the review and report any limitations of the synthesis and how they may affect the conclusions.

## Discussion

Differences in health systems organization, structure and resources will determine the need and success of task-shifting interventions for DR screening. The review will examine how these interventions have been used and/or tested in LMICs. This rapid review will provide context-specific evidence to inform policy makers and programme managers tasked with designing and implementing services to prevent and manage diabetes and its complications in low- and middle- income settings.

In balancing the time demands of a rapid review methodology, we have prioritized the peer-reviewed literature as we considered the more robust evidence for effectiveness will be found in published scientific journals. We acknowledge that the limits in searches and time may potentially miss some evidence which could have been published before the period included and in the grey literature.

## Supplementary Information

**Additional file 1.** Reporting standards – PRISMA-P Checklist.

**Additional file 2.** Registration OSF: https://osf.io/h5wgr/

**Additional file 3.** Search strategy.

## Data Availability

Not applicable—no data sets currently available.

## Abbreviations

DR: Diabetic retinopathy
LMIC: Low- and middle-income country
SSA: Sub-Saharan Africa
PLWD: People living with diabetes
HIC: High-income country
WHO: World Health Organization
DTA: Diagnostic test accuracy
OCT: Ocular coherence tomography
OSF: Open Science Framework
PRISMA-P: Preferred Reporting Items for Systematic Reviews and Meta-Analysis Protocols
SING: Scottish Intercollegiate Guidelines Network
EDTRS: Early Treatment Diabetic Retinopathy Study
CPD: Continuing professional development
SWiM: Synthesis without meta-analysis
GRADE: Grading of Recommendations Assessment, Development and Evaluation
