# Supplementary material for: Effectiveness of task-shifting for the detection of diabetic retinopathy in low- and middle-income countries: a rapid review protocol
Source: Syst Rev. 2021 Jan 4;10:4. doi: 10.1186/s13643-020-01553-w (PMC7780379; doi:10.1186/s13643-020-01553-w)
Supplement: Supplementary file 2 — Additional file 2. Registration OSF: https://osf.io/h5wgr/ [file 13643_2020_1553_MOESM2_ESM.docx]

**Additional File 2: Protocol Registration details**

This protocol was reg**istered** on OSF on 29-04-2020

Citation:

Bascaran, Covadonga, Nyawira Mwangi, Fabrizio D’Esposito, Shaffi Mdala, Juan A L Ulloa, Iris Gordon, Jacqueline Ramke, Jennifer Evans, and Matthew Burton. 2020. “Effectiveness of Task-Shifting for the Detection of Diabetic Retinopathy in Low- and Middle-Income Countries: A Rapid Review Protocol.” OSF. May 13. osf.io/dfhg6.

Identifier:

DOI 10.17605/OSF.IO/DFHG6
