# Supplementary material for: Effectiveness of task-shifting for the detection of diabetic retinopathy in low- and middle-income countries: a rapid review protocol
Source: Syst Rev. 2021 Jan 4;10:4. doi: 10.1186/s13643-020-01553-w (PMC7780379; doi:10.1186/s13643-020-01553-w)
Supplement: Supplementary file 3 — Additional file 3. Search strategy. [file 13643_2020_1553_MOESM3_ESM.docx]

**Additional File 3: Search strategy**

**Effectiveness of task-shifting for the detection of diabetic retinopathy in low- and middle-income countries: a rapid review protocol.**

Covadonga Bascaran^1^, Nyawira Mwangi^1,2^, Fabrizio D’Esposito^5^, Iris Gordon^1^, Juan Alberto Lopez Ulloa^4^, Shaffi Mdala^3^, Jacqueline Ramke^1^, Jennifer R Evans^1^, Matthew Burton^1,6^

Affiliation

^1^ London School of Hygiene and tropical Medicine, London, United Kingdom

^2^ Kenya Medical Training College, Nairobi, Kenya

^3^ Queen Elizabeth Central Hospital, Blantyre, Malawi

^4^ Centro Mexicano de Salud Visual Preventiva, Ciudad de México

^5^ The Fred Hollows Foundation, Melbourne, Australia

^6^ Moorfields Eye Hospital, London, UK

^7^ School of Optometry and Vision Science, University of Auckland, Auckland, New Zealand

**Medline Ovid search strategy**

1. exp Diabetes Mellitus/

2. exp Diabetes Complications/

3. Diabetic Retinopathy/

4. ((diabet$ or proliferative or non-proliferative) adj4 retinopath$).tw.

5. diabetic retinopathy.kw.

6. (diabet$ adj3 (eye$ or vision or visual$ or sight$)).tw.

7. (retinopath$ adj3 (eye$ or vision or visual$ or sight$)).tw.

8. (DR adj3 (eye$ or vision or visual$ or sight$)).tw.

9. or/1-8

10. exp Mass Screening/

11. exp Vision Tests/

12. exp Telemedicine/

13. exp Photography/

14. exp Ophthalmoscopes/

15. exp Ophthalmoscopy/

16. (ophthalmoscop$ or fundoscop$ or funduscop$).ti.

17. ((exam$ or photo$ or imag$) adj3 fundus).tw.

18. (photography or retinography).tw.

19. ((mydriatic or digital or retina$ or fundus or steroscopic) adj3 camera).tw.

20. ((mydriatic or digital or retina$ or fundus or steroscopic) adj3 imag$).tw.

21. screen$.tw.

22. diagnos$.tw.

23. detection.tw.

24. ((eye$ or retina$ or ophthalm$) adj4 exam$).tw.

25. ((eye or vision or retinopathy or ophthalmic) adj4 test$).tw.

26. ((eye$ or retina$ or ophthalm$) adj4 visit$).tw.

27. Office Visits/

28. (telemedicine$ or telemonitor$ or telescreen$ or telehealth or teleophthalmology).tw.

29. or/10-28

30. (task$ adj2 (shift$ or shar$)).tw.

31. (ophthalmic adj3 (physician$ or nurse$ or technician$ or officer$ or assistant$ or staff$ or worker$)).tw.

32. (eye$ adj3 (physician$ or nurse$ or technician$ or officer$ or assistant$ or staff$ or worker$)).tw.

33. (healthcare adj2 profession$).tw.

34. (allied adj2 health adj2 worker$).tw.

35. ((physician$ or medical) adj2 assistant$).tw.

36. Optometrists/

37. (optometrist$ or optician$ or orthopist$ or refractionists).tw.

38. (community adj3 (aide$ or worker$)).tw.

39. (worker$ adj3 (voluntary or volunteer$ or lay village$)).tw.

40. (lay adj2 (worker$ or person$)).tw.

41. or/30-40

42. 9 and 29 and 41

43. limit 42 to yr="2010 - 2020"

44. limit 43 to english language

45. exp case reports/

46. (case adj2 report$).tw.

47. or/45-46

48. 44 not 47

49. limit 48 to (comment or editorial or letter or news)

50. 48 not 49

**Embase OVID search strategy**

1. exp diabetes mellitus/di, dm, ep, pc [Diagnosis, Disease Management, Epidemiology, Prevention]

2. exp diabetic retinopathy/

3. ((diabet$ or proliferative or non-proliferative) adj4 retinopath$).tw.

4. diabetic retinopathy.kw.

5. (diabet$ adj3 (eye$ or vision or visual$ or sight$)).tw.

6. (retinopath$ adj3 (eye$ or vision or visual$ or sight$)).tw.

7. (DR adj3 (eye$ or vision or visual$ or sight$)).tw.

8. or/1-7

9. exp Screening/

10. exp Vision Test/

11. Eye Examination/

12. Telemedicine/

13. Photography/

14. Eye Photography/

15. Ophthalmoscopy/

16. (ophthalmoscop$ or fundoscop$ or funduscop$).ti.

17. ((exam$ or photo$ or imag$) adj3 fundus).tw.

18. (photography or retinography).tw.

19. ((mydriatic or digital or retina$ or fundus or steroscopic) adj3 camera).tw.

20. ((mydriatic or digital or retina$ or fundus or steroscopic) adj3 imag$).tw.

21. screen$.tw.

22. diagnos$.tw.

23. detection.tw.

24. ((eye$ or retina$ or ophthalm$) adj4 exam$).tw.

25. ((eye or vision or retinopathy or ophthalmic) adj4 test$).tw.

26. ((eye$ or retina$ or ophthalm$) adj4 visit$).tw.

27. (telemedicine$ or telemonitor$ or telescreen$ or telehealth or teleophthalmology).tw.

28. or/9-27

29. (task$ adj2 (shift$ or shar$)).tw.

30. (ophthalmic adj3 (physician$ or nurse$ or technician$ or officer$ or assistant$ or staff$ or worker$)).tw.

31. (eye$ adj3 (physician$ or nurse$ or technician$ or officer$ or assistant$ or staff$ or worker$)).tw.

32. (healthcare adj2 profession$).tw.

33. (allied adj2 health adj2 worker$).tw.

34. ((physician$ or medical) adj2 assistant$).tw.

35. Optometrists/

36. (optometrist$ or optician$ or orthopist$ or refractionists).tw.

37. (community adj3 (aide$ or worker$)).tw.

38. (worker$ adj3 (voluntary or volunteer$ or lay village$)).tw.

39. (lay adj2 (worker$ or person$)).tw.

40. or/29-39

41. 8 and 28 and 40

42. limit 41 to yr="2010 -Current"

43. limit 42 to english language

44. exp case report/

45. (case adj2 report$).tw.

46. or/44-45

47. 43 not 46

48. limit 47 to (conference abstract or conference paper or "conference review" or editorial or letter or note)

49. 47 not 48

**Global Health OVID search strategy**

1. diabetes/

2. ((diabet$ or proliferative or non-proliferative) adj4 retinopath$).tw.

3. (diabet$ adj3 (eye$ or vision or visual$ or sight$)).tw.

4. (retinopath$ adj3 (eye$ or vision or visual$ or sight$)).tw.

5. (DR adj3 (eye$ or vision or visual$ or sight$)).tw.

6. or/1-5

7. screening/

8. screen$.tw.

9. diagnos$.tw.

10. detection.tw.

11. ophthalmoscopy/

12. (ophthalmoscop$ or fundoscop$ or funduscop$).ti.

13. ((exam$ or photo$ or imag$) adj3 fundus).tw.

14. (photography or retinography).tw.

15. ((mydriatic or digital or retina$ or fundus or steroscopic) adj3 camera).tw.

16. ((mydriatic or digital or retina$ or fundus or steroscopic) adj3 imag$).tw.

17. ((eye$ or retina$ or ophthalm$) adj4 exam$).tw.

18. ((eye or vision or retinopathy or ophthalmic) adj4 test$).tw.

19. ((eye$ or retina$ or ophthalm$) adj4 visit$).tw.

20. telemedicine/

21. (telemedicine$ or telemonitor$ or telescreen$ or telehealth or teleophthalmology).tw.

22. or/7-21

23. (task$ adj2 (shift$ or shar$)).tw.

24. (ophthalmic adj3 (physician$ or nurse$ or technician$ or officer$ or assistant$ or staff$ or worker$)).tw.

25. (eye$ adj3 (physician$ or nurse$ or technician$ or officer$ or assistant$ or staff$ or worker$)).tw.

26. (healthcare adj2 profession$).tw.

27. (allied adj2 health adj2 worker$).tw.

28. ((physician$ or medical) adj2 assistant$).tw.

29. (optometrist$ or optician$ or orthopist$ or refractionists).tw.

30. (community adj3 (aide$ or worker$)).tw.

31. (worker$ adj3 (voluntary or volunteer$ or lay village$)).tw.

32. (lay adj2 (worker$ or person$)).tw.

33. or/23-32

34. 6 and 22 and 33

35. limit 34 to yr="2010 -Current"

36. limit 35 to english language

37. case reports/

38. (case adj2 report$).tw.

39. or/37-38

40. 36 not 39

41. limit 40 to (conference or conference paper or conference proceedings or correspondence or editorial)

42. 40 not 41
